# Pregnancy and maternal glomerular disease: a closer look

**DOI:** 10.1590/2175-8239-JBN-2025-0299en

**Published:** 2026-02-06

**Authors:** Muhammad Taha Naeem, Saad Ahmed

**Affiliations:** 1 University of Health Science, Quaid e Azam Medical College, Bahawalpur, Pakistan.

Dear Editor,

We have read with great interest the article by Marques et al. titled “The effects of pregnancy on the progression of maternal glomerular disease”^1^. The study provides significant insights into maternal and fetal risks among women with glomerular disease (GD). However, several methodological concerns warrant attention.

This research highlights chronic hypertension (CH) as a risk factor for adverse outcomes. However, the results suggest that CH did not directly lead to an accelerated decline in glomerular filtration rate (GFR). Instead, CH primarily raised the likelihood of developing superimposed preeclampsia, which was responsible for the GFR loss. This establishes an indirect relationship. Prior studies also emphasize that hypertensive disorders affect renal outcomes chiefly through endothelial and podocyte damage linked to preeclampsia^2^.

Additionally, the decision to exclude diabetic nephropathy is noteworthy. Diabetic nephropathy represents the leading global cause of chronic kidney disease (CKD) and is closely related to complications during pregnancy. Pregnant women with diabetic CKD face a higher risk of preeclampsia, premature delivery, and the need for neonatal intensive care compared to those with diabetes but without CKD^3^.

Moreover, the retrospective design raises concerns about reliability. Retrospective cohorts are prone to recall and report biases since outcomes are predetermined. In contrast, prospective designs enable standardized data collection and more robust causal conclusions. This distinction is especially vital for high-risk populations such as pregnant women with CKD^4^.

Finally, the lack of a control group complicates the attribution of adverse effects solely to GD. Conditions such as pre-eclampsia, pre-term labor, and low birth weight can also occur in otherwise healthy pregnancies. Including comparison groups would strengthen the findings and clarify whether the results are specifically related to GD^5^.

In summary, Marques et al.^1^ provide important insights into pregnancy in women with GD. However, future studies should (i) clarify direct versus indirect risk mechanisms, (ii) include patients with diabetic nephropathy, (iii) utilize prospective study designs, and (iv) incorporate control groups.

## Data Availability

This manuscript is a Letter to the Editor and does not involve the generation or analysis of original research data. As a result, no datasets were created or examined.
