# Supplementary material for: Pregnancy and maternal glomerular disease: a closer look
Source: J Bras Nefrol. 2026 Feb 6;48(1):e20250299. doi: 10.1590/2175-8239-JBN-2025-0299en (PMC12884835; doi:10.1590/2175-8239-JBN-2025-0299en)
Supplement: Translation

# Translation: Gravidez e doença glomerular materna: um olhar mais atento

**DOI:** 10.1590/2175-8239-JBN-2025-0299pt

**Authors:** Muhammad Taha Naeem, Saad Ahmed

**Affiliations:** 1 University of Health Science, Quaid e Azam Medical College, Bahawalpur, Pakistan.

Prezado Editor,

Lemos com grande interesse o artigo de Marques et al. intitulado “Os efeitos da gravidez na progressão da doença glomerular materna”^1^. O estudo fornece informações significativas sobre os riscos maternos e fetais entre mulheres com doença glomerular (DG). No entanto, diversas questões metodológicas merecem atenção.

Esta pesquisa destaca a hipertensão crônica (HC) como um fator de risco para desfechos adversos. No entanto, os resultados sugerem que essa condição não levou diretamente a um declínio acelerado na taxa de filtração glomerular (TFG). Em vez disso, a HC aumentou principalmente a probabilidade de desenvolvimento de pré-eclâmpsia sobreposta, responsável pela perda da TFG, estabelecendo-se assim uma relação indireta. Estudos anteriores também enfatizam que os distúrbios hipertensivos afetam os desfechos renais principalmente por meio de lesões endoteliais e podocitárias associadas à pré-eclâmpsia^2^.

Além disso, a decisão de excluir a nefropatia diabética merece destaque. A nefropatia diabética representa a principal causa global de doença renal crônica (DRC) e está intimamente relacionada a complicações durante a gravidez. Gestantes com DRC diabética enfrentam maior risco de pré-eclâmpsia, parto prematuro e necessidade de terapia intensiva neonatal em comparação àquelas com diabetes, mas sem DRC^3^.

Ademais, o desenho retrospectivo levanta preocupações quanto à confiabilidade. Coortes retrospectivas são propensas a vieses de memória e relato, uma vez que os desfechos são predeterminados. Em contrapartida, desenhos prospectivos permitem a coleta padronizada de dados e conclusões causais mais robustas. Essa distinção é especialmente vital para populações de alto risco, como gestantes com DRC^4^.

Por fim, a ausência de um grupo controle dificulta a atribuição dos efeitos adversos exclusivamente à DG. Condições como pré-eclâmpsia, parto prematuro e baixo peso ao nascer também podem ocorrer em gestações saudáveis. A inclusão de grupos de comparação fortaleceria os achados e ajudaria a esclarecer se os resultados estão especificamente relacionados à DG^5^.

Em resumo, Marques et al.^1^ fornecem informações importantes sobre a gestação em mulheres com DG. No entanto, estudos futuros devem (i) esclarecer os mecanismos de risco diretos versus indiretos; (ii) incluir pacientes com nefropatia diabética; (iii) utilizar desenhos prospectivos; e (iv) incorporar grupos controle.

## Data Availability

Este manuscrito é uma Carta ao Editor e não envolve a geração ou análise de dados de pesquisa originais. Como resultado, nenhum conjunto de dados foi criado ou analisado.
